# Exploring the spatiotemporal relationship between influenza and air pollution in Fuzhou using spatiotemporal weighted regression model

**DOI:** 10.1038/s41598-024-54630-8

**Published:** 2024-02-19

**Authors:** Qingquan Chen, Xiaoyan Zheng, Binglin Xu, Mengcai Sun, Quan Zhou, Jin Lin, Xiang Que, Xiaoyang Zhang, Youqiong Xu

**Affiliations:** 1 The Affiliated Fuzhou Center for Disease Control and Prevention of Fujian Medical University, Fuzhou, 350005 China; 2 The School of Public Health, Fujian Medical University, Fuzhou, 350108 China; 3 China Resources Double Crane Pharmaceutical Co Ltd, Beijing, 100079 China; 4 Fujian Agriculture and Forestry University, Fuzhou, 350028 China

**Keywords:** Influenza, Spatiotemporal weighted regression, Air pollutant, Spatial heterogeneity, Dynamic time warping, K-medoids algorithms, Respiratory tract diseases, Environmental sciences, Medical research

## Abstract

Air pollution has become a significant concern for human health, and its impact on influenza, has been increasingly recognized. This study aims to explore the spatiotemporal heterogeneity of the impacts of air pollution on influenza and to confirm a better method for infectious disease surveillance. Spearman correlation coefficient was used to evaluate the correlation between air pollution and the influenza case counts. VIF was used to test for collinearity among selected air pollutants. OLS regression, GWR, and STWR models were fitted to explore the potential spatiotemporal relationship between air pollution and influenza. The R^2^, the RSS and the AICc were used to evaluate and compare the models. In addition, the DTW and K-medoids algorithms were applied to cluster the county-level time-series coefficients. Compared with the OLS regression and GWR models, STWR model exhibits superior fit especially when the influenza outbreak changes rapidly and is able to more accurately capture the changes in different regions and time periods. We discovered that identical air pollutant factors may yield contrasting impacts on influenza within the same period in different areas of Fuzhou. NO_2_ and PM_10_ showed opposite impacts on influenza in the eastern and western areas of Fuzhou during all periods. Additionally, our investigation revealed that the relationship between air pollutant factors and influenza may exhibit temporal variations in certain regions. From 2013 to 2019, the influence coefficient of O_3_ on influenza epidemic intensity changed from negative to positive in the western region and from positive to negative in the eastern region. STWR model could be a useful method to explore the spatiotemporal heterogeneity of the impacts of air pollution on influenza in geospatial processes. The research findings emphasize the importance of considering spatiotemporal heterogeneity when studying the relationship between air pollution and influenza.

## Introduction

Influenza is an acute respiratory disease caused by the influenza virus (IV), which is a class C infectious disease in China^1^. The clinical manifestations of influenza are mainly high fever, fatigue, headache, cough, systemic muscle soreness and other systemic disease symptoms, while respiratory symptoms are mild. Sudden outbreaks and rapid spread cause different degrees of epidemics and are the most significant epidemiological features of influenza^2^. According to WHO estimates, annual seasonal influenza epidemics can cause 3 to 5 million severe cases and 290,000 to 650,000 deaths related to respiratory diseases worldwide^3^.As a result, it has become an important public health issue^4^.

The epidemic characteristics of influenza in China are different in the north and south. The peak of influenza in northern China mostly occurs in the cold winter and spring, while that in southern China occurs all year, and peaks mostly occur in winter and summer. However, this is not absolute. For example, Jinan and Tibet have also had small influenza peaks in summer^5^. An outbreak of influenza will lead to public panic and social and economic depression and seriously affect social stability and healthy development^6^. Influenza transmission risk factors encompass individual immune susceptibility^7^, population mobility, meteorological conditions (e.g., low temperatures and reduced ultraviolet radiation)^8^, and air pollution^9^.

Many previous studies have consistently demonstrated a significant correlation between air pollution and influenza incidence. Su W et al. used wavelet coherence analysis and a generalized Poisson superimposed regression model to study the potential relationship between air pollutant and influenza-like illness (ILI) in Jinan, China, from 2016 to 2017 and found that air pollutant, especially PM_2.5_, PM_10_, CO, and SO_2_, could increase the risk of ILI^10^. Pascal M et al. found an interaction between temperature and PM_10_ on respiratory diseases and mortality^11^. However, not all air pollutant will accelerate influenza. McGee Hargrove M et al. found that high concentrations of O_3_ can kill influenza virus in the air or on the surface of objects to reduce the spread of influenza without harm to humans^12^. Song et al. utilized Moran’s I and correlation analysis to examine the spatiotemporal differentiation characteristics of influenza incidence in prefecture-level cities and explore its relationship with air pollution^13^. However, in these studies, linear regression models or spatial measurement models were used to explore the relationship between influenza and air pollution, but these methods often ignore the temporal heterogeneity of air pollution on influenza.

Geographically weighted regression (GWR) model is an effective spatial statistical model that accommodates the spatial non-stationarity of relationships between studied factors and their influencing factors by considering local heterogeneity in space^14^. Ibarra-Zapata E et al. used GWR model to explore the spatial non-stationarity of Influenza type A and its influencing factors in Mexico^15^. Although GWR provides a more precise and location-specific analysis of spatial patterns, it does not capture spatio-temporal synergies. Considering that the relationship between influenza and its influencing factors may have significant spatial and temporal variation, targeted research on the specific correlation between regional influenza cases and air pollution may provide a reference and countermeasures to judge the regional and seasonal changes in influenza, improve the capacity of atmospheric environmental governance and reduce the rapid spread of the influenza virus. Spatiotemporal geographically weighted regression (STWR) model, a spatiotemporal regression model, incorporates the heterogeneity in the relationship between variables^16^. In comparison to GWR and geographically and temporally weighted regression (GTWR) models, STWR model performs better in analyzing and explaining local spatiotemporal nonstationarity. This is achieved through clarifying the concept of "time distance" and introducing novel temporal kernel and spatiotemporal kernel functions based on this concept.Therefore, STWR model was utilized to examine the spatial and temporal variation relationship between influenza and air pollution.

This study aims to conduct a descriptive analysis of the epidemiological characteristics of influenza cases in Fuzhou and construct OLS regression, GWR, and STWR models to investigate the spatiotemporal heterogeneity of influenza at the county level in Fuzhou. Additionally, the impact of air pollution on influenza may vary over time. By determining the optimal regression model, we will comprehensively explain the spatiotemporal heterogeneity of the impacts of different air pollutants on influenza incidence. The findings of this study will provide scientific evidence for the prevention and control strategies of influenza at the county-level in Fuzhou and support in response to future influenza outbreaks.

## Methods

### Study area

**Figure 1:**
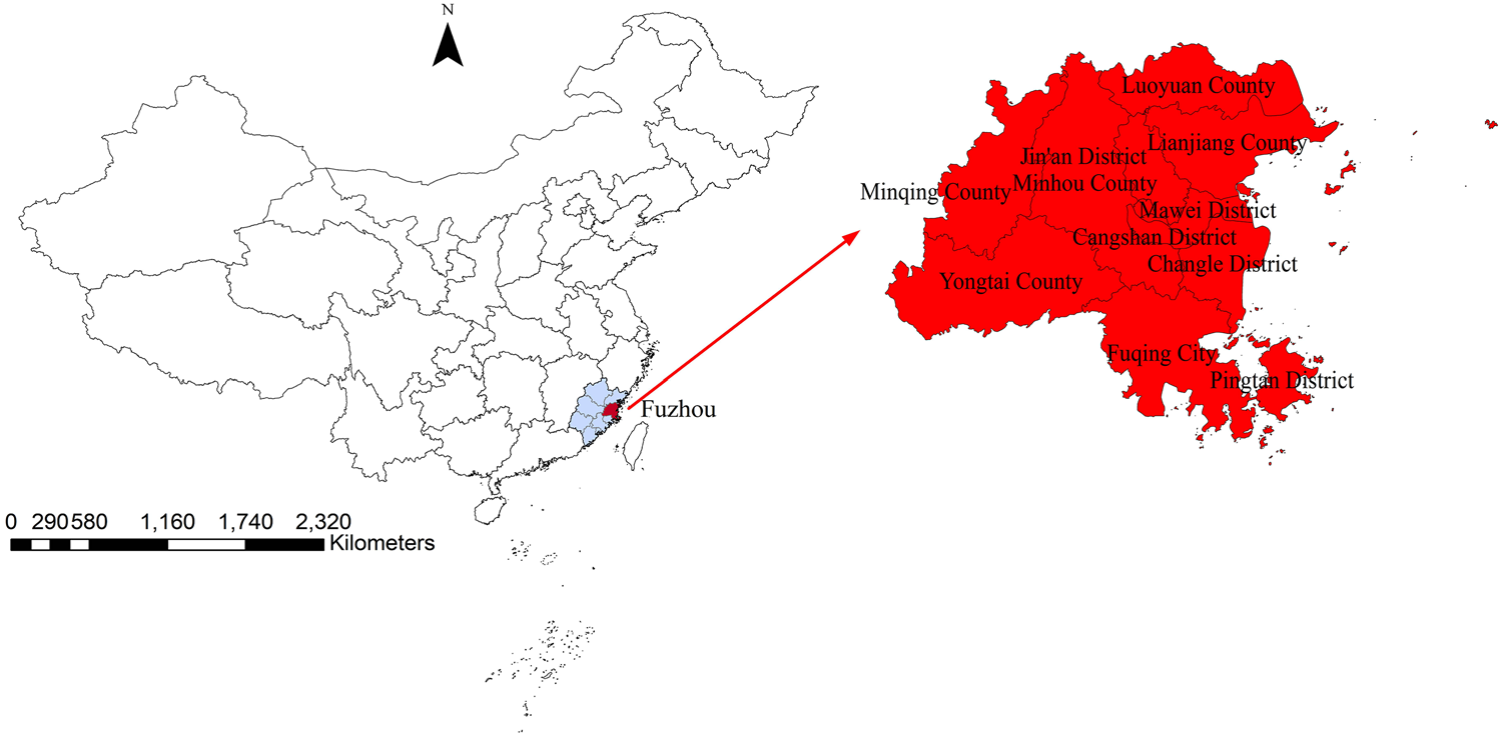
Location of Fuzhou City, China.

As a coastal city in southeast China, the provincial capital city of Fujian Province, Fuzhou, consists of six districts, one county-level city and six counties^12^. The population in Fuzhou has increased from 7.12 million to 8.24 million in the past decade. The geographical location of Fuzhou is depicted in Fig. 1.

### Data source

#### Influenza case data

The Chinese Nationwide Notifiable Infectious Diseases Reporting Information System (CNIDRIS) was implemented in 2004 and covers all healthcare institutions throughout China. Since its implementation, legally reported infectious disease cases have been promptly reported within this system. Currently, the system includes a total of 40 infectious diseases. The data regarding influenza cases in Fuzhou from 2013 to 2019 were obtained from the CNIDRIS. We utilized solely anonymized aggregated data, which excluded sensitive information of cases, including names, valid identification numbers, work units, and contact numbers. In the final dataset, only essential information, such as gender, age, occupation, administrative region of residence, diagnosis date, and onset date, remained. The above data were classified and analyzed based on the administrative region of residence.

#### Ethical considerations

The ethical research board committee of Fuzhou Center for Disease Control and Prevention (Approval No. IRB2020008) approved the research. The need for individual informed consents is waived by the institution/ review board due to the face that exclusively utilized anonymized aggregated data was used and did not involve any individual subjects. This study was carried out following the Helsinki Declaration contents.

#### Air pollution data

The monthly air pollution data for the period from January 2013 to December 2019 used in this study primarily originated from the China Air Quality Reanalysis Data Set (CAQRA). CAQRA was generated through a collaboration between the Institute of Atmospheric Physics, Chinese Academy of Sciences (IAP/CAS), the Chinese National Center for Environmental Monitoring (CNEMC), and other research institutions (https://doi.org/10.11922/sciencedb.00053). It provides a surface grid dataset for six air pollutant (PM_2.5_, PM_10_, SO_2_, NO_2_, CO, and O_3_) at a spatial resolution of 15 km.

### Statistical analysis

Descriptive statistics were employed to illustrate the characteristics of the population distribution and temporal patterns of influenza. The incidence of influenza per 100,000 population in each year was calculated by dividing the influenza case counts by the population of that year. The time trend of the incidence of influenza was analyzed using the Cochran-Armitage trend test. Histograms and line charts were utilized to show the temporal distribution of influenza. ArcGIS (version 10.5; ESRI) was used to depict the geographical distribution of influenza cases. Spearman's correlation coefficient was employed to evaluate the correlation between factors of air pollution and influenza case counts. The variance inflation factor (VIF) was used to examine collinearity among the selected air pollutants. Data management and statistical analysis were conducted using SPSS (version 26; IBM Corp) and R (version 4.2.1; The R Foundation).

### Regression analysis

We fit OLS regression, GWR, and STWR models to explore the potential spatiotemporal relationship between air pollution and influenza. The three models were fit by using the F-STWR 2.1.5^17^. By comparing the performance of these models, we wanted to determine which one can more accurately predict and explain the impact of air pollution on influenza. Smoothed maps of Ordinary Kriging were employed in ArcGIS (version 10.5; ESRI) to illustrate the spatial pattern and impacts of various air pollutant on the influenza epidemic level. Furthermore, we employed the dynamic temporal regularity (DTW) and K-medoids algorithms to assess the county-level impacts and characteristics of air pollution on influenza. These algorithms were used to cluster the time series of county-level coefficients. Classifying the coefficients in different counties provides valuable insights into understanding the transmission and influencing factors of influenza in various geographical areas.

### Ordinary least squares

Ordinary least squares (OLS) regression model, a traditional linear regression model, is utilized to estimate the association between independent and dependent variables by minimizing the sum of squared residuals^18^. We employed the OLS regression model to investigate the global relationship between air pollutants and influenza case counts in Fuzhou. The model can be presented by Eq. (1).

1 $$y={\beta }_{0}+\sum_{k=1}^{p}{\beta }_{k}{x}_{k}+\varepsilon$$

where $y$ is the influenza case counts, ${\beta }_{0}$ is the model’s intercept, ${x}_{k}$ corresponds to the $k$th air pollutants variable of the model ($k$= 1 to $p$), and $\varepsilon$ is the random error. Being a global model, OLS regression assumes a uniform relationship between independent and dependent variables across the entire study area. Consequently, it does not consider the analysis of localized regional characteristics.

### Geographically weighted regression

Geographically weighted regression (GWR) model builds upon OLS regression model by incorporating the spatial location of the data, allowing the regression coefficients to vary based on geographical location^19^. In this study, we employed the GWR model to investigate the local spatial relationship between air pollutants and influenza case counts in Fuzhou. In Eq. (2), the sample's position (u, v) is introduced into the regression equation to estimate local parameters. The model accounts for the spatial heterogeneity that exists between the independent and dependent variables.

2 $${y}_{i}={\beta }_{0}\left({u}_{i},{v}_{i}\right)+\sum_{k=1}^{p}{\beta }_{k}{\left({u}_{i},{v}_{i}\right)x}_{ik}+{\varepsilon }_{i}$$

where ${y}_{i}$ is the influenza case counts for location $i$, ${u}_{i}$ and ${v}_{i}$ are the coordinates of location $i$, ${\beta }_{0}\left({u}_{i},{v}_{i}\right)$ is the intercept at location $i$, ${\beta }_{k}\left({u}_{i},{v}_{i}\right)$ is the local parameter estimate for air pollutants variable ${x}_{ik}$ at location $i$, and ${\varepsilon }_{i}$ is the error term.

For predicting the regression coefficients of GWR model, the distance-decay function (${w}_{ij}$) is employed as a weighted factor that considers the distance between the modeled positions and the observed values. When the distribution of sampling points is irregular, an adaptive weight function is used to adjust the bandwidth based on the density of the spatial points, as depicted in Eq. (3).

3 $$w_{ij} = \left\{ {\begin{array}{*{20}c} {\left[ {1 - \left( {d_{ij} /b} \right)^{2} } \right]^{2} } & {d_{ij} \le b} \\ 0 & {d_{ij} > b} \\ \end{array} } \right.$$

where ${d}_{ij}$ is the distance between observation $i$ and $j$, $b$ is the adaptive bandwidth. For a case in which the distance between observations is greater than the adaptive bandwidth, the distance-decay function becomes zero. GWR model utilizes neighboring points surrounding each observation point as weights, thus reflecting the varying degrees of influence from different geographical locations. Consequently, GWR model can more accurately capture and account the spatial heterogeneity in the influenza case counts across different regions in Fuzhou.

### Ethics approval and consent

The ethical research board committee of Fuzhou Center for Disease Control and Prevention (Approval No. IRB2020008) approved the research. The need for individual informed consents is waived by the institution/ review board due to the face that exclusively utilized anonymized aggregated data was used and did not involve any individual subjects.

## Spatiotemporal weighted regression

Spatiotemporal weighted regression (STWR) proposes a new numerical time-varying decay weighting strategy and adopts a new spatiotemporal kernel for analysing processes that contain both spatial and temporal heterogeneity. In STWR, the time distance is the rate of change of the attribute value within a time interval, rather than the time interval itself^20^. STWR is the comprehensive time-varying numerical difference rate information in the time interval on the basis of GWR. Its basic calculation framework is consistent with that of GWR^21^. We utilized the STWR model to explore the local spatiotemporal relationship between air pollutants and influenza case counts in Fuzhou. The model can be expressed as:

4 $${y}_{i}^{t}={\beta }_{0}^{t}\left({u}_{i},{v}_{i}\right)+{\sum }_{k}{\beta }_{k}^{t}({u}_{i},{v}_{i}){x}_{ik}^{t}+{\varepsilon }_{i}^{t}$$

In Eq. (4), ${{\text{y}}}_{i}^{t}$ represents the influenza case counts of the $t$ period, the *i*^th^ regression point regression point $({u}_{i},{v}_{i})$, ${\varepsilon }_{i}^{t}$ is the random error term that satisfies air pollutants and identical distribution, and ${\beta }_{0}^{t}\left({u}_{i},{v}_{i}\right)$ and ${\beta }_{k}^{t}({u}_{i},{v}_{i})$ represent the constant term and coefficient of the $t$ period and the *i*^th^ regression point $({u}_{i},{v}_{i})$, respectively^15^. The calculation formula is:

5 $${\widehat{\beta }}^{t}\left({u}_{i},{v}_{i}\right)={[{(X}_{{S}_{\Delta t}}^{T}{W}_{\Delta t}\left({u}_{i},{v}_{i}\right){X}_{{S}_{\Delta t}})}^{-1}{X}_{{S}_{\Delta t}}{W}_{\Delta t}({u}_{i},{v}_{i})]{y}_{{S}_{\Delta t}}^{T}$$

In Eq. (5), ${X}_{{S}_{\Delta t}}$ is the ground matrix of local air pollutants observed in the time interval $\Delta t$, and ${W}_{\Delta t}\left({u}_{i},{v}_{i}\right)$ are the space–time weight matrices of observed values in different positions and time periods. Its subelement ${W}_{ij}$ represents the influence of the $j$ observation point on the $i$ regression point, which can be calculated according to the kernel function according to the distance.

The time interval decay weight assignment strategies of Gaussian, bisquare and GTWR are usually different. STWR uses a time assignment function based on the numerical difference rate between the regression point and the observation point^22^. The weighted average form of the spatiotemporal kernel in STWR is given by Eq. (6).

6 $$W_{ij\Delta t}^{t} = \left\{ {\begin{array}{*{20}c} {\left[ {\frac{2}{{1 + exp\left( { - \frac{{\left| {\left( {y_{i\left( t \right)} - y_{{j\left( {t - q} \right)}} } \right)/y_{{j\left( {t - q} \right)}} } \right|}}{{\Delta t/b_{T} }}} \right)}} - 1} \right],} & {if\; 0 < \Delta t < b_{T} } \\ {0,} & {otherwise} \\ \end{array} } \right.$$

In Eq. (6), ${y}_{i\left(t\right)}-{y}_{j\left(t-q\right)}$ represents the numerical difference between the regression point in $i$ and $j$ in $t-q$ within time interval $\Delta t$; ${b}_{T}$ is the time bandwidth. This assignment function can more effectively capture the different time influence weights of the historical observation point on the regression point.

## Comparison of OLS regression, GWR and STWR models

The average R-squared (R^2^) is an indicator used to measure the degree of fitness of a regression model to observed data, representing the proportion of variance in the dependent variable that can be explained by the model. The residual sum of squares (RSS) is a metric that measures the fitting error of the model, indicating the degree of difference between the predicted values and the actual observed values. The corrected Akaike information criterion (AICc) is an indicator of the relative information loss in the model estimation process, taking into account the model's goodness of fit and the number of parameters. Therefore, a better-performing model has a higher R^2^ value and lower RSS and AICc values. By comparing R^2^, RSS, and AICc, we can evaluate and compare the performance of OLS regression, GWR, and STWR models.

## Dynamic time warping and K-medoids algorithm

Dynamic time warping (DTW) constructs the correspondence of two sequence elements of different lengths according to the principle of proximity and evaluates the similarity of two sequences^23^. It is widely applied in the assessment of time-series similarity. Meanwhile, it is also considered to be the most accurate method to evaluate the similarity of time-series data^24^. The calculation method of DTW is given by Eq. (7).

7 $$DTW=\left|{x}_{i}-{y}_{j}\right|+min\left\{D\left(i-1,j\right),D\left(i,j-1\right),D\left(i-1,j-1\right)\right\}$$

where ${x}_{i}$ and ${y}_{j}$ represent the values of each graph and *D* represents the distance between two points. Through $\left|{x}_{i}-{y}_{j}\right|$, the difference between the two sequences is first measured, and then the minimum number in the previous values is added.

The DTW algorithm is usually used in conjunction with the K-medoids algorithm, which is a partitioning-based clustering algorithm^25^. The K-medoids algorithm is an unsupervised machine learning technique that is able to effectively partition the observations in the dataset into different clusters with a centre for each cluster^26^. The K-medoids algorithm steps are performed in the following order: (1) randomly select K samples as centres, (2) calculate the distance of all samples to randomly selected K centres, (3) assign the samples to the nearest centre, (4) mark them as a group, (5) calculate the total distance and so-called total cost, and (6) repeat these steps until the lowest total cost is obtained.

## Research workflow

**Figure 2:**
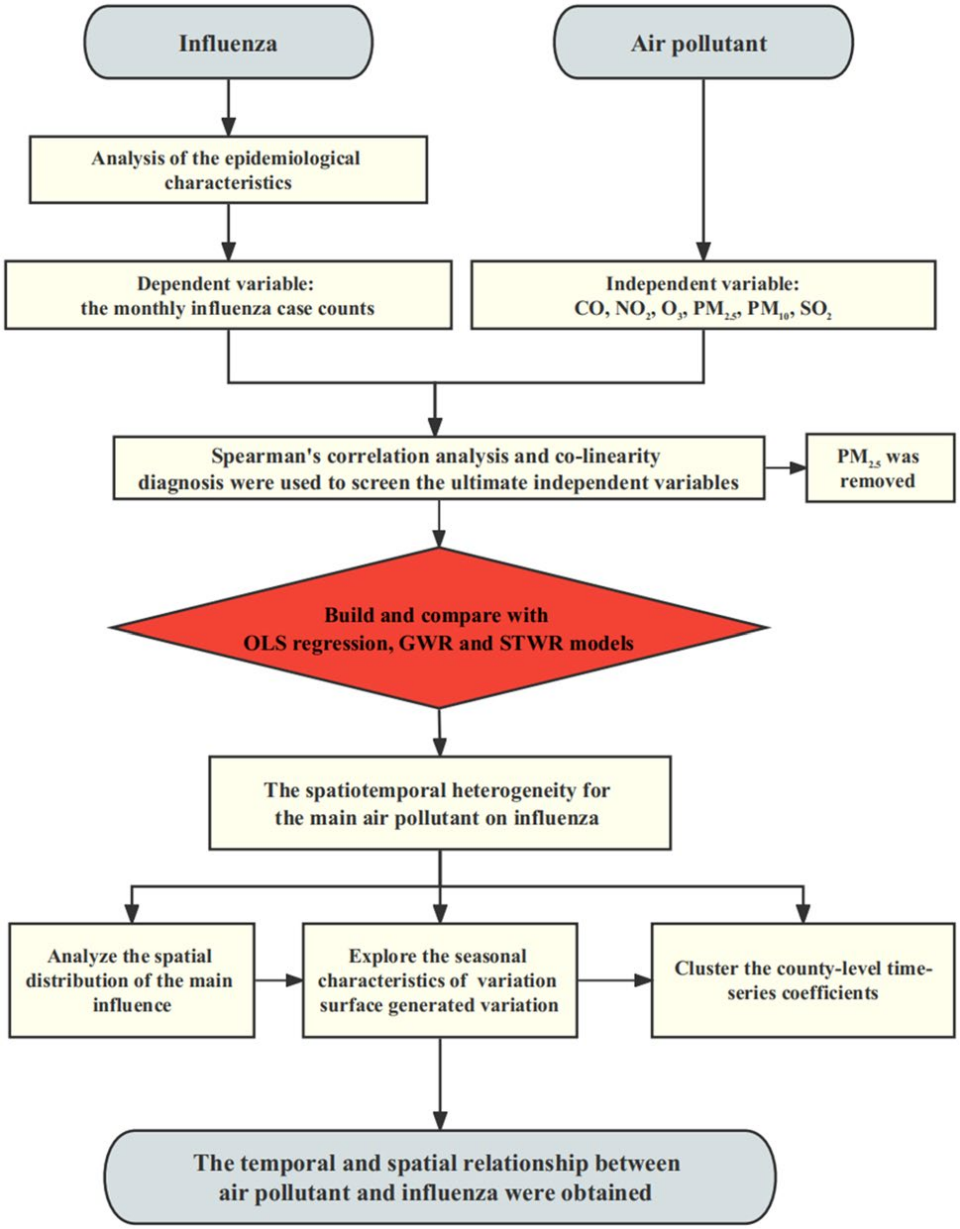
Research workflow.

Figure 2 shows the research workflow in this study. First, we collected the monthly influenza case counts in Fuzhou from 2013 to 2019 and described the epidemiological characteristics. Second, Spearman's correlation analysis and collinearity diagnosis were used to screen the ultimate air pollutants. Both the screened variables and the dependent variable were then normalized. Third, OLS regression, GWR and STWR models were constructed. Fourth, the spatial coefficient of variation surface generated by STWR model was used to explore and analyse the spatiotemporal heterogeneity of various air pollutant on the influenza epidemic level. Spatiotemporal heterogeneity refers to the variation or differences in the distribution and occurrence of a phenomenon (in this study, influenza case counts) across both space and time. Finally, the DTW and K-medoids algorithms were applied to cluster the county-level time-series coefficients.

## Results

### Epidemiological characteristics

There were 11,524 influenza cases reported in Fuzhou between 2013 and 2019, with an average annual incidence of 20.54 cases per 100,000 people, including 6,518 male cases and 5,006 female cases. The under 14 years old group accounted for 68.74% of all the reported cases and had the highest incidence rate of 559.43 cases per 100,000 people. Among all occupational groups, Scattered children accounted for the largest proportion (37.50%) of cases, followed by others (27.87%) and students (23.72%). The demographic characteristics of influenza cases in Fuzhou from 2013 to 2019 are shown in Table 1.

**Table 1:** Demographic characteristics of influenza cases in Fuzhou, China 2013–2019.

	2013	2014	2015	2016	2017	2018	2019	Total
Incidence	12.71	16.25	16.08	13.23	12.70	24.42	48.36	20.54
Gender								
Male	542 (56.11)	659 (52.34)	716 (57.1)	602 (57.83)	576 (56.25)	1124 (56.34)	2299 (57.69)	6518 (56.56)
Female	424 (43.89)	600 (47.66)	538 (42.9)	439 (42.17)	448 (43.75)	871 (43.66)	1686 (42.31)	5006 (43.44)
Sex ratio	1.28	1.1	1.33	1.37	1.29	1.29	1.36	1.30
Age group								
0–14	799 (82.71)	826 (65.61)	915 (72.97)	595 (57.16)	584 (57.03)	1376 (68.97)	2827 (70.94)	7922 (68.74)
15–59	130 (13.46)	355 (28.2)	276 (22.01)	354 (34.01)	313 (30.57)	433 (21.7)	823 (20.65)	2684 (23.29)
≥ 60	37 (3.83)	78 (6.2)	63 (5.02)	92 (8.84)	127 (12.4)	186 (9.32)	335 (8.41)	918 (7.97)
Occupation								
Kindergarten children	42 (4.35)	64 (5.08)	56 (4.47)	39 (3.75)	66 (6.45)	324 (16.24)	666 (16.71)	1257 (10.91)
Scattered children	717 (74.22)	626 (49.72)	643 (51.28)	374 (35.93)	283 (27.64)	637 (31.93)	1041 (26.12)	4321 (37.50)
Student	62 (6.42)	214 (17)	247 (19.7)	264 (25.36)	255 (24.9)	454 (22.76)	1238 (31.07)	2734 (23.72)
Others	145 (15.01)	355 (28.2)	308 (24.56)	364 (34.97)	420 (41.02)	580 (29.07)	1040 (26.1)	3212 (27.87)
Total	966	1259	1254	1041	1024	1995	3985	11,524

Incidence is the average annual incidence rate (per 100,000 populations).

**Figure 3:**
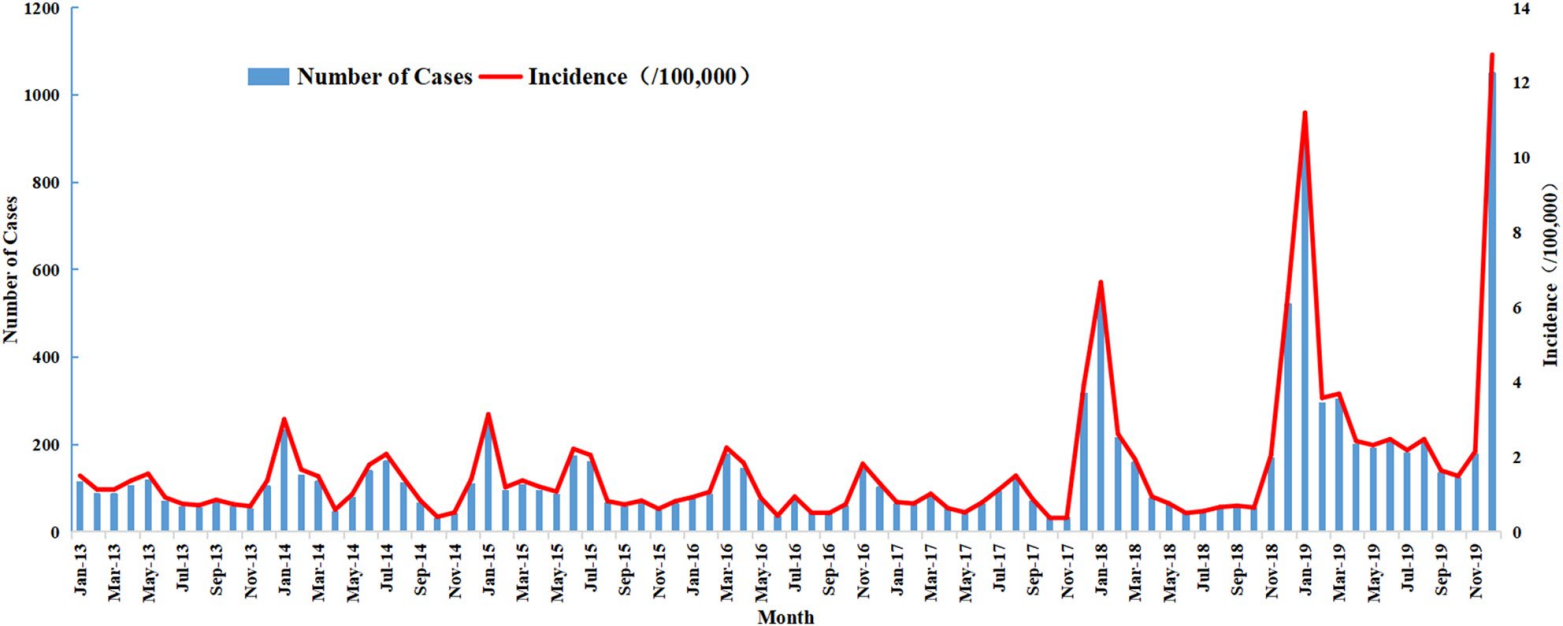
Monthly incidence and reported cases of influenza in Fuzhou, China 2013–2019.

During the 7-year study period, there was an ascending long-term trend (*Z* = 45.055, *P* < 0.001) in the incidence of influenza in Fuzhou, and the highest annual incidence rate was in 2019 (48.36 cases per 100,000 people). Meanwhile, there was significant seasonal variation in the monthly distribution of influenza cases in Fuzhou, with two significant peaks from April to July and from November to February (Fig. 3). Moreover, the number of cases during the second peak (59.97%) was usually greater than that during the first peak (23.3%).

**Figure 4:**
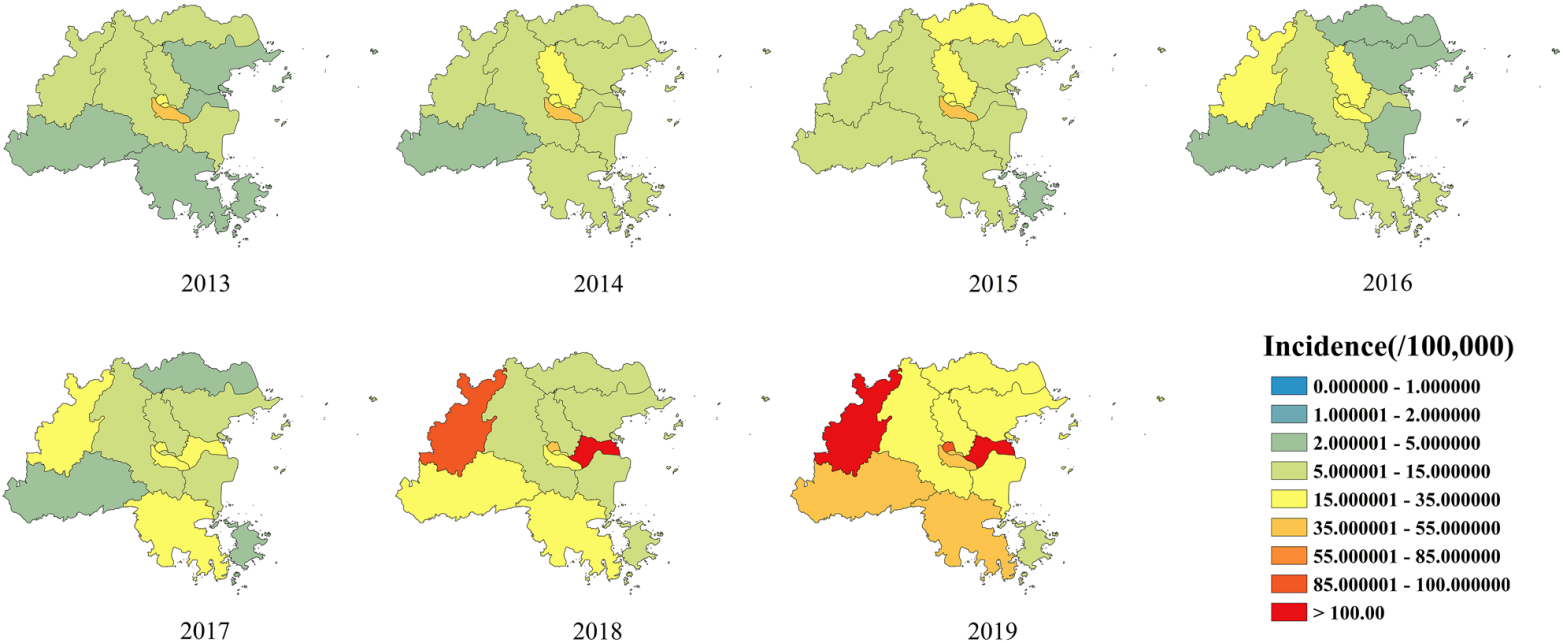
Geographic distribution of influenza incidence in Fuzhou, China 2013–2019.

Figure 4 demonstrates the incidence of influenza in each district and county in Fuzhou from 2013 to 2019. In the past seven years, influenza cases have been reported in all the 13 areas of Fuzhou. In general, the incidence of influenza in central Fuzhou was generally higher than that in the surrounding areas during 2013–2015. Since 2016, the high incidence of influenza in Fuzhou has extended from the central region to the surrounding areas, among which Minqing County and Mawei District became the main areas with a high incidence of influenza (Fig. 4).

### Spearman's correlation analysis and collinearity diagnostics

As shown in Table 2, all air pollutants were significantly related to influenza case counts. There is a negative correlation between O_3_ and influenza case count (Spearman's correlation coefficient < 0), while the other air pollutant show a positive correlation with influenza case count (Spearman's correlation coefficient > 0). The correlations between NO_2_ and influenza were significantly higher compared to other air pollutant, with a maximum value of 0.499.

**Table 2:** Spearman's correlation results between influenza case counts and air pollutant in Fuzhou, China 2013–2019.

Year	CO	NO_2_	O_3_	PM_2.5_	PM_10_	SO_2_
2013	0.426^a^	0.499^a^	-0.327^a^	0.245^a^	0.369^a^	0.188^a^
2014	0.130	0.408^a^	-0.347^a^	0.205^a^	0.275^a^	0.281^a^
2015	0.207^a^	0.346^a^	-0.147	0.229^a^	0.323^a^	0.201^a^
2016	0.315^a^	0.484^a^	-0.148	0.313^a^	0.412^a^	0.274^a^
2017	0.162^a^	0.265^a^	-0.113	0.200^a^	0.235^a^	0.405^a^
2018	0.326^a^	0.464^a^	-0.369^a^	0.199^a^	0.143	0.386^a^
2019	0.280^a^	0.378^a^	-0.264^a^	0.283^a^	0.230^a^	0.256^a^

^a^The significance level (*P* value) of correlation coefficient is < 0.05.

Since all the absolute values of Spearman's correlation coefficients were below 0.6, a subsequent multicollinearity test was performed for the six air pollutant. PM_2.5_ was removed, ensuring that the VIFs of the remaining air pollutant remained below 10 (Table 3).

**Table 3:** Colinearity diagnostics of air pollutant in Fuzhou, China 2013–2019.

Year	2013	2014	2015	2016	2017	2018	2019
CO	6.966	1.902	4.211	4.151	3.936	4.806	2.795
NO_2_	6.407	6.077	5.314	6.248	6.033	5.395	8.548
O_3_	2.743	1.920	2.249	2.443	1.577	1.880	3.759
PM_10_	6.195	3.989	8.270	7.368	4.301	3.085	7.990
SO_2_	2.951	4.506	4.572	2.937	2.691	2.780	2.268

### Analysis of spatiotemporal heterogeneity of influenza and air pollutant

#### Comparison of model performances

We compared the performance of the STWR model with OLS regression and GWR models (Table 4). In comparison to the results of OLS regression and GWR models, STWR model showed the highest R^2^, indicating a better fit. Additionally, STWR model exhibited the lowest RSS value among the three models, suggesting a superior fit. Similar conclusions were drawn for the AICc value. Overall, STWR model emerges as a more favorable choice for the scope of this study.

**Table 4:** Performance comparison of OLS regression, GWR and STWR models.

Model	R^2^	RSS	AICc	Sigma
OLS regression	0.513	6.338	53.006	NA
GWR	0.639	4.692	105.299	0.961
STWR	0.816	2.389	−296.125	0.647

**Figure 5:**
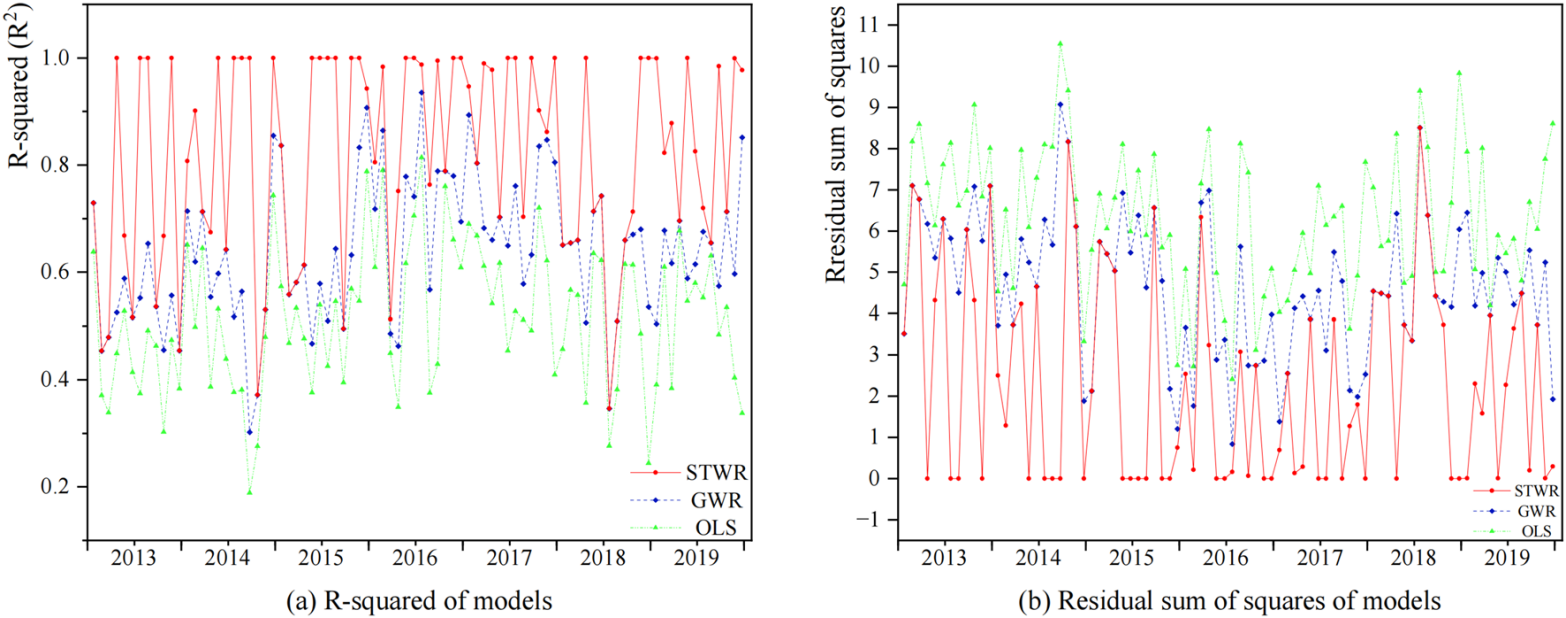
(**a**) R-squared values of the models and (**b**) the residual sum of squares values of the models from 2013 to 2019.

Further comparisons were made for the monthly R^2^ and RSS values. Compared to OLS regression and GWR models, STWR model consistently maintained the highest R^2^ and lowest RSS across the entire study period (Fig. 5). This further highlights the superior fitting performance of STWR model in this study.

**Figure 6:**
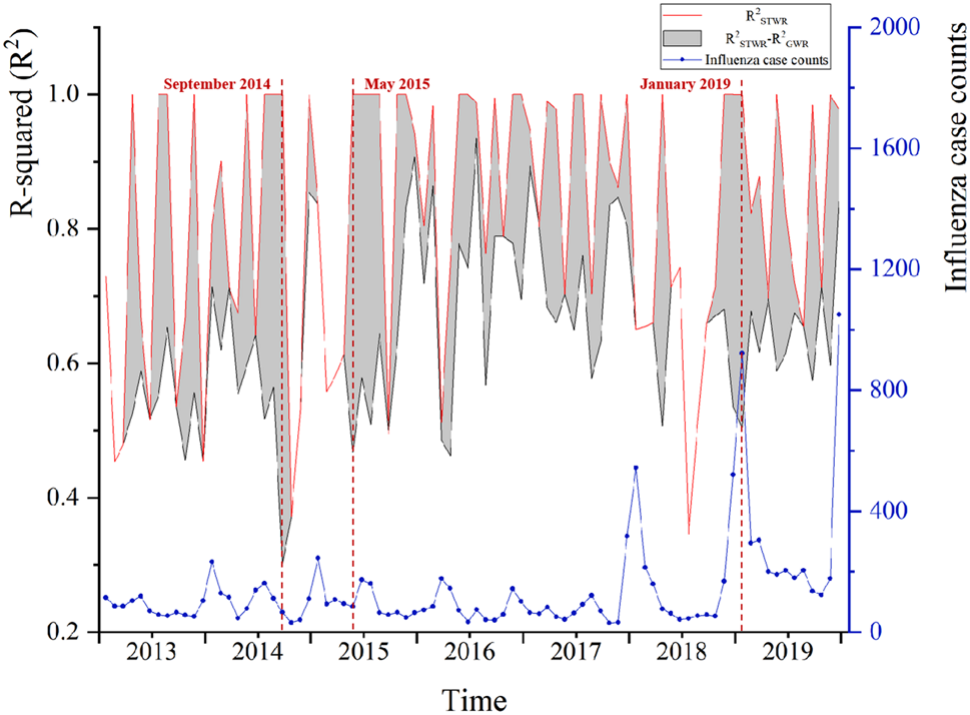
Comparison of R^2^ difference of GWR and STWR models with the influenza case counts.

The fit of STWR model is affected by the influenza case counts. The three largest differences in R^2^ values between STWR and GWR models were observed in September 2014, May 2015, and January 2019 (Fig. 6). These time points generally corresponded to influenza peak seasons and periods of increased influenza activity throughout the study period. The superior fitting performance of the STWR model becomes more pronounced in the presence of rapid changes in the influenza case counts.

#### Influenza epidemic level variation of spatial coefficient corresponding to different air pollutant

**Figure 7:**
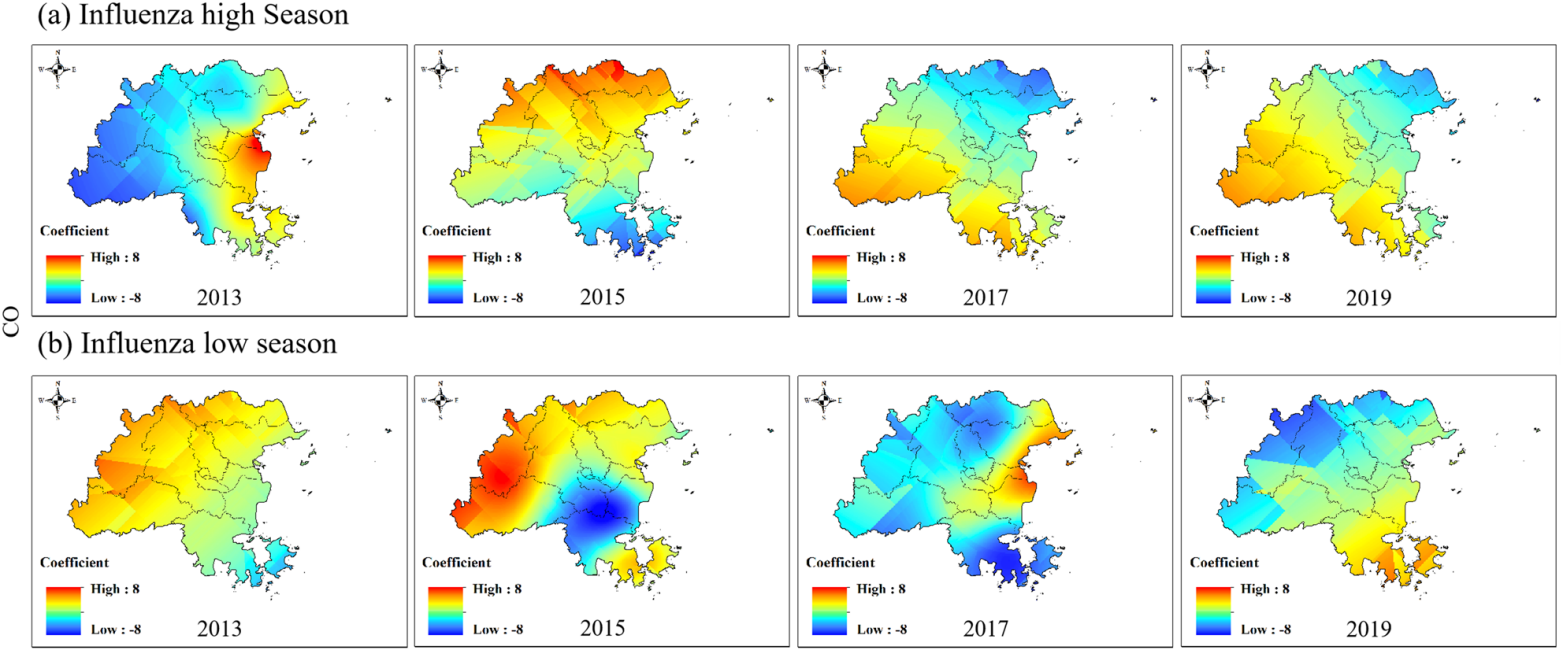
Spatial variation coefficient surface of the impact of CO on influenza in Fuzhou.

To explore the spatial distribution of the impacts of different air pollutant on the influenza epidemic level variation in Fuzhou, one year was divided into the influenza high season (e.g., top 6 ranking for influenza case counts) and influenza low season (e.g., bottom 6 ranking for influenza case counts) according to the actual influenza epidemiological data in Fuzhou. Figure 7 showed that the negative impact of CO on the western regional influenza epidemic gradually became positive over time during the peak influenza season. This means that the higher the CO concentration is, the greater the epidemic intensity of influenza. In the influenza low season, the impact of CO on the northwest region gradually changed from positive to negative. This means that the higher the CO concentration is, the lower the epidemic intensity of influenza (Fig. 7 and Figure S1).

**Figure 8:**
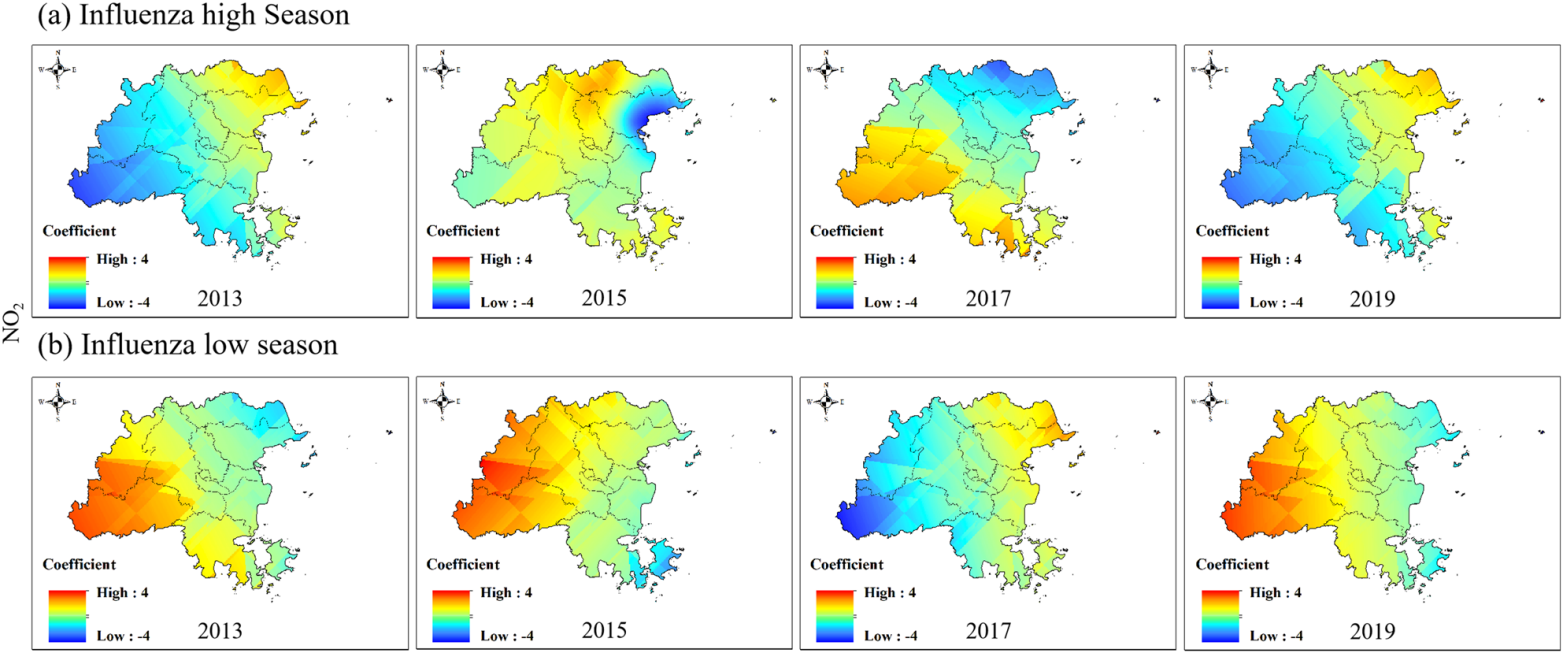
Spatial variation coefficient surface of the impact of NO_2_ on influenza in Fuzhou.

The impact of NO_2_ on the influenza epidemic was always the opposite in the eastern and western regions of Fuzhou (Fig. 8 and Figure S2). In the influenza high season, the positive impact of NO_2_ on the influenza epidemic in the western region of Fuzhou gradually changed from 2013 to a negative impact in 2017 and eventually returned to a positive impact in 2019. The eastern region experienced the opposite impact. In the low influenza season, NO_2_ had a positive impact on the influenza epidemic in the western region of Fuzhou but showed a negative impact in 2017.

**Figure 9:**
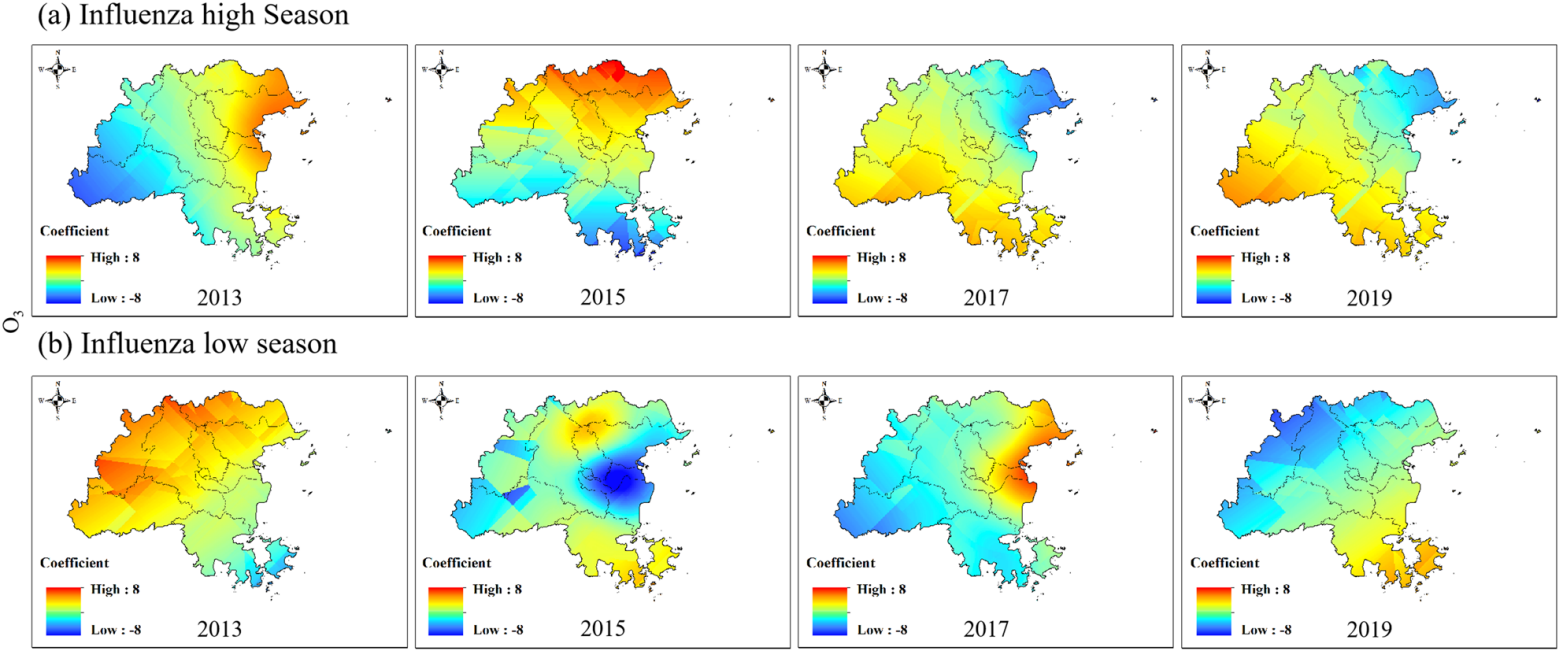
Spatial variation coefficient surface of the impact of O_3_ on influenza in Fuzhou.

Consistent with CO, the influence coefficient of O_3_ on the influenza epidemic changed from negative to positive in the western region and from positive to negative in the eastern region during the influenza high season (Fig. 9 and Figure S3). In the influenza low season of 2015, O_3_ showed a strong negative impact near Taijiang District, and high concentrations of O_3_ could reduce the intensity of influenza.

**Figure 10:**
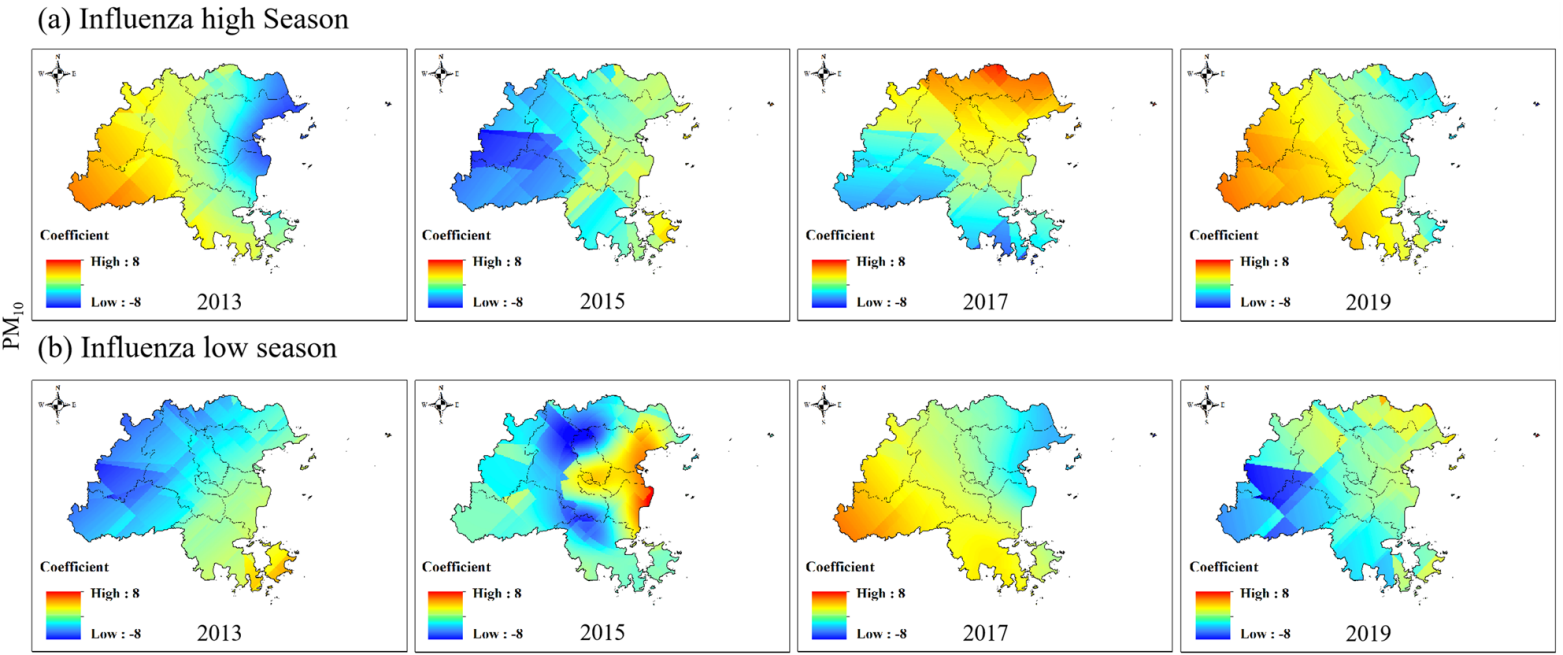
Spatial variation coefficient surface of the impact of PM_10_ on influenza in Fuzhou.

Compared with NO_2_, the influence coefficient of PM_10_ on the influenza epidemic was also always opposite in the eastern and western regions of Fuzhou (Fig. 10 and Figure S4). The impact coefficient changed from positive and negative to positive during the influenza high season in the western region of Fuzhou. In the influenza low season, the opposite impact was observed, i.e., from positive to negative to positive again.

**Figure 11:**
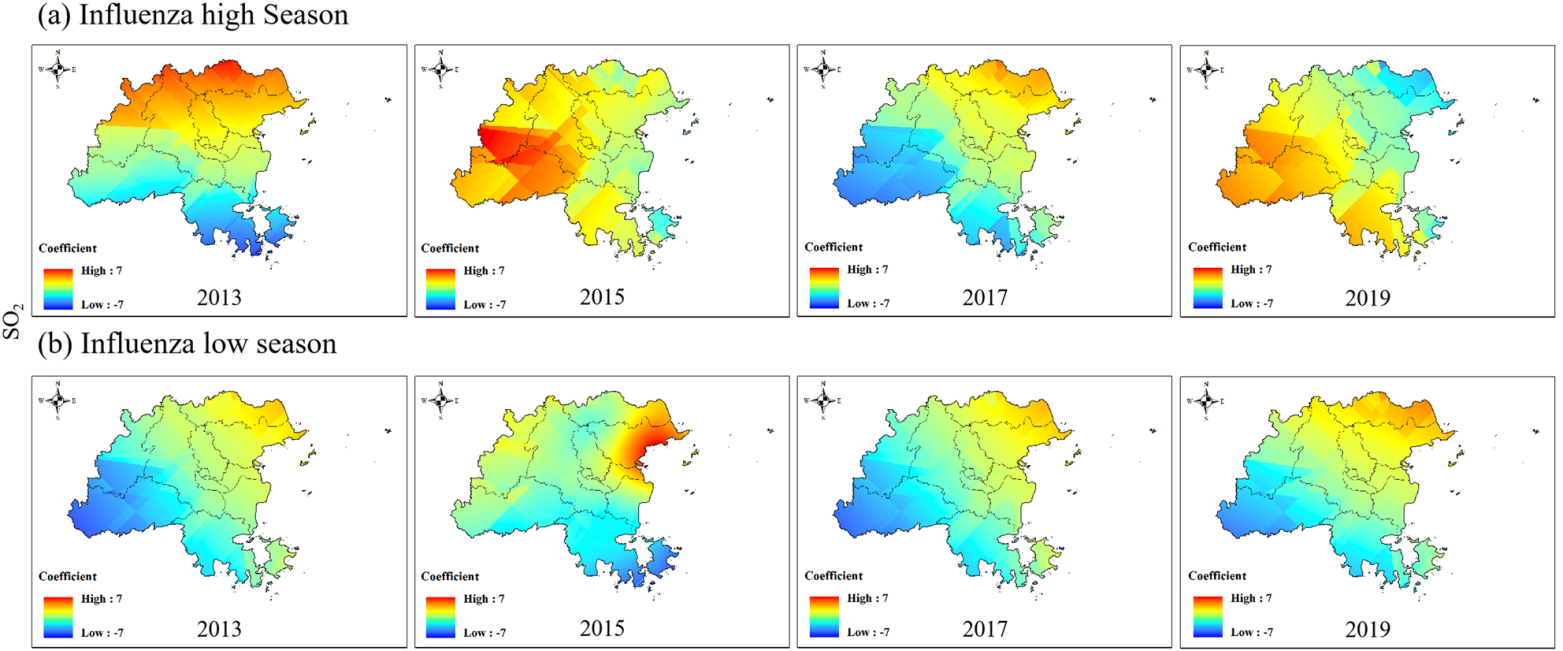
Spatial variation coefficient surface of the impact of SO_2_ on influenza in Fuzhou.

As shown in Fig. 11 and Figure S5, the impact coefficient of SO_2_ on the influenza epidemic was basically consistent in all areas of Fuzhou in 2017 and the low influenza season in 2013 and 2019. In the influenza high season of 2015, SO_2_ had a positive impact on the influenza epidemic near Minqing County, and the high concentration of SO_2_ may have increased the intensity of the influenza epidemic. In contrast, during the influenza low season, SO_2_ had a negative impact near Yongtai County.

#### Cluster the county-level time-series coefficients

We used the DTW algorithm to assess the similarity of county-level time-series coefficients to further investigate the coefficient surfaces of geospatial processes. The K-medoids algorithm was then used to cluster the districts and counties according to similarity. The optimal "K" was determined to be 4 by using the elbow method. Finally, the time-series coefficients of the four cluster centres can be aggregated monthly to form a heatmap.

**Figure 12:**
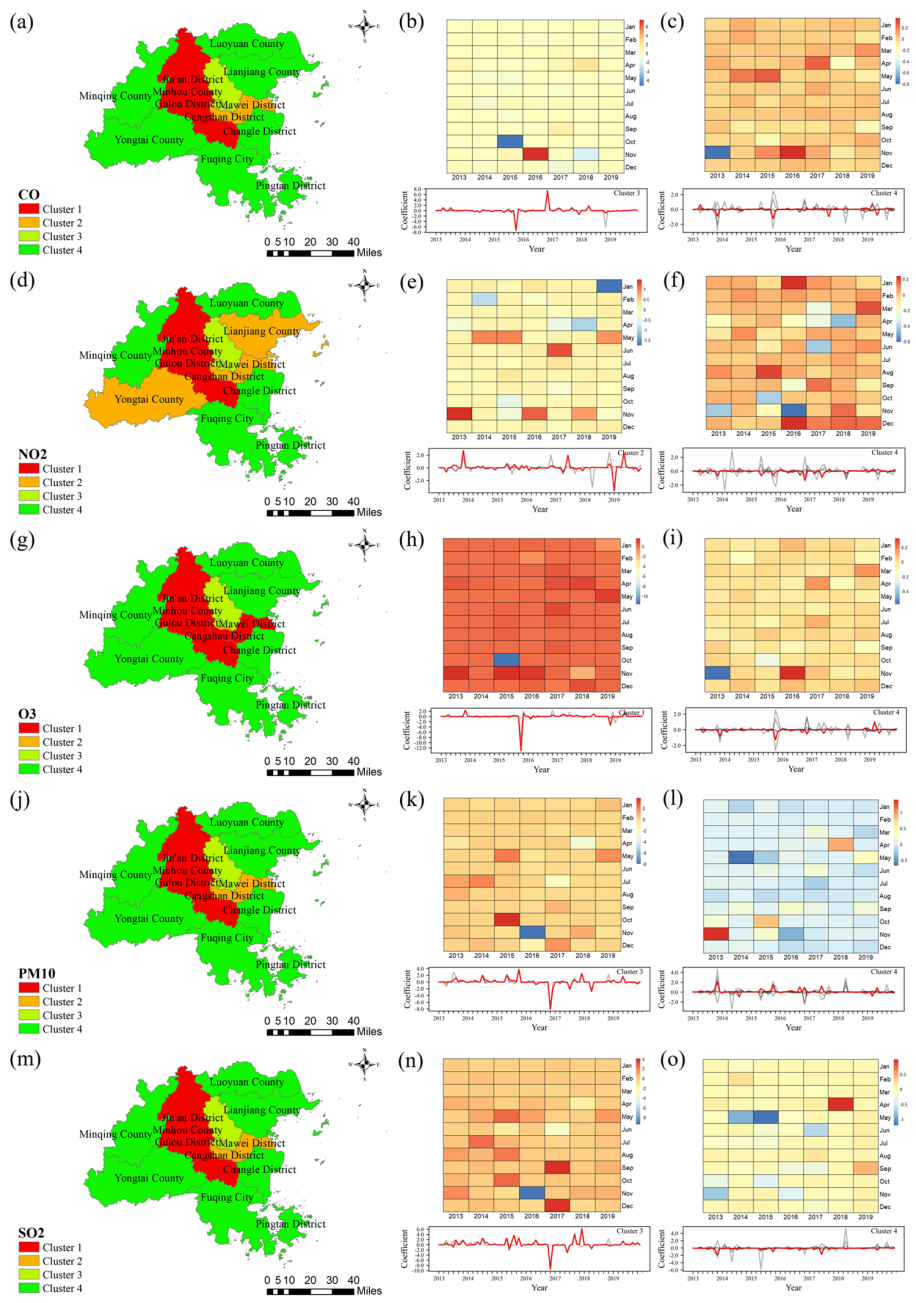
Time series clustering results of the coefficients of CO, NO_2_, O_3_, PM_10_, and SO_2_ on influenza. (**a**, **d**, **g**, **j**, **m**) show the spatial distribution of county-level clustering results for CO, NO_2_, O_3_, PM_10_, and SO_2_, respectively. (**b**, **c**) are the time series plots and heat plots of CO coefficients in the centre of Cluster 3 and Cluster 4, respectively. (**e**, **f**) are the time series plots and heat plots of NO_2_ coefficients in the centre of Cluster 3 and Cluster 4, respectively. (**h**, **i**) are the time series plots and heat plots of O_3_ coefficients in the centre of Cluster 1 and Cluster 4, respectively. (**k**, **l**) are the time series plots and heat plots of PM_10_ coefficients in the centre of Cluster 3 and Cluster 4, respectively. (**n**, **o**) are the time series plots and heat plots of SO_2_ coefficients in the centre of Cluster 3 and Cluster 4, respectively.

The time-series results of the impact of CO on the influenza epidemic were clustered (Fig. 12a), Jin'an District, Gulou District and Taijiang District formed a group (Cluster 3), and CO had a positive impact on the influenza epidemic from January to September (Fig. 12b). The higher the CO concentration is, the greater the intensity of the influenza epidemic. Minqing County, Yongtai County, Fuqing City and Luoyuan County were grouped into Cluster 4. The CO in this region had a greater positive impact on influenza epidemics during most of the period (Fig. 12c).

The clustering results of NO_2_ showed that Cluster 2 was composed of Cangshan District, Mawei District, Yongtai County and Lianjiang County (Fig. 12d). The concentration of NO_2_ increased in the summer and fall, which may increase the intensity of the influenza epidemic. In particular, NO_2_ in May and November was also higher than that in other months, with average influence coefficients of 0.3988 and 0.4010, respectively. However, the NO_2_ in Cluster 2 had a significant negative impact on the influenza epidemic in January 2019 (Fig. 12e). In addition, the areas around Fuzhou City, such as Minqing County, Luoyuan County and Changle District, formed Cluster 4. It had obvious positive impacts in winter (Fig. 12f). The mean coefficients of variation in December and January were 0.0918 and 0.0428, respectively.

O_3_ aggravated the intensity of influenza epidemics in some areas (Fig. 12h), such as in Cangshan District, Minhou County and Mawei District (Cluster 1) (Fig. 12g). Simultaneously, it also showed a relatively small positive impact on Cluster 4, i.e., Minqing County, Yongtai County, Fuqing City and Luoyuan County (Fig. 12i). The clustering results were identical for PM_10_ and SO_2_ (Fig. 12j, m). Cluster 3 included Jin'an District, Gulou District and Taijiang District, with PM_10_ and SO_2_ generally having positive impacts on the influenza epidemic (Fig. 12k, n). For Cluster 4, which was composed of the surrounding areas of Fuzhou, PM_10_ had a negative impact on the influenza epidemic, while SO_2_ showed a positive impact on the influenza epidemic (Fig. 12l, o).

## Discussion

### Epidemiological characteristics of influenza

Since 2013, the epidemic of influenza in Fuzhou has shown an obvious trend of high incidence in winter and spring, which is similar to the monitoring situation in Hangzhou and Guangzhou in China^27^. The incidence of influenza in Fuzhou is increasing yearly, especially in 2019, a seasonal H3N2 influenza outbreak^28^, indicating that the prevention and control of influenza in Fuzhou is still in a severe situation. Influenza has become one of the key infectious diseases for prevention and control in Fuzhou. Every outbreak of influenza has caused great economic loss to the Fuzhou area and poses a serious threat to people's health. The central urban area of Fuzhou is a region with a high incidence of influenza, mainly because of the dense population in the central urban area, with a large migrant population, poor living conditions and high living density, and poor awareness of disease prevention. Scattered children are the main group affected by influenza. This phenomenon may be related to the living environment and behavioral habits of scattered children. Scattered children usually reside in different areas and have more contact with people, making them more susceptible to potential influenza viruses. In addition, scattered children may be more likely to spread the virus in schools or social settings because they have more frequent contact with other children.

### Comparison of the fitting impacts of OLS regression, GWR, and STWR models

OLS regression model is a global regression model that assumes a fixed relationship between variables^29^. GWR model is a local regression model that considers the spatial non-stationarity of the relationship between variables^30^. STWR model is a spatiotemporal regression model that considers the temporal heterogeneity of the relationship between variables. STWR model utilizes different regression coefficients at various spatiotemporal points to better explain the changing relationship between variables under different temporal and spatial conditions^20^. Therefore, STWR model can make more full use of the critical time-varying information of history to improve model performance, which provides more accurate prediction model and analytical statistical method for spatiotemporal epidemiological studies of infectious diseases such as influenza.

We explored the relationship between the influenza case counts and major air pollution from 2013 to 2019 through OLS regression, GWR and STWR models. Through comparing R_2_, RSS, and AICc, we found that STWR model had the best goodness of fit compared with OLS regression and GWR (Table 4, Figs. 5, 6). Meanwhile, the STWR model fitting results also had a significant advantage when the influenza case counts changed rapidly.

### Spatiotemporal heterogeneity in the impacts of air pollution on influenza

In recent years, air pollution has become an important public health problem worldwide, and extensive epidemiological and clinical evidence shows that short-term and long-term exposure to air pollutant will increase the incidence risk and mortality of many systemic diseases, such as cardiovascular, cerebrovascular and respiratory diseases^31,32^. Karen et al. examined the impact of air pollution on the total population and infant mortality in the United States during the 1918 influenza pandemic^33^. The study found that the severity of air pollution is related to the urban coal-fired power generation capacity. The study results found that air pollution exacerbated the pandemic. Compared with low coal cities, high coal city infant mortality increased by 11%, medium coal city increased by 8%, and whole population mortality increased by 10% and 5%. Results from Australia showed that increasing PM_10_ and O_3_ concentrations will increase paediatric influenza cases, with impact RR values of 1.11 (1.10–1.13) and 1.28 (1.25–1.31), respectively^34^. Santus et al. studied the association between atmospheric pollutants and respiratory diseases and found that every 1 mg/m^3^ increase in CO increased the number of emergency cases of upper respiratory tract infection between 0–5 d^35^.

In our study, we conducted a spatiotemporal regression analysis using STWR model to explore the relationship between air pollution and influenza. By incorporating the geographic location and temporal information of county-level region in Fuzhou, our analysis reflected for the spatiotemporal heterogeneity in the impacts of air pollution on influenza. The results demonstrate significant variations in the impact of air pollution on the influenza epidemic level between county-level areas and different time (Figs. 7, 8, 9, 10, 11).

We also found that the same air pollution in the same influenza pandemic period may have two completely opposite impacts in different regions. For example, the impacts of PM_10_ and NO_2_ on influenza epidemics were always opposite in the eastern and western regions of Fuzhou (Figs. 8, 10). SO_2_ occasionally exhibited similar results as well. The eastern and western regions of Fuzhou may have different sources of pollution, leading to variations in the composition and characteristics of PM_10_, NO_2_ and SO_2_. These differences can result in varying impacts on the influenza epidemic in each region. The meteorological conditions, such as wind patterns and atmospheric stability, can differ between the eastern and western regions of Fuzhou. These conditions can influence the dispersion and accumulation of PM_10_, NO_2_ and SO_2_, thereby affecting their impact on the influenza epidemic. The eastern and western regions of Fuzhou may have different population densities and behavioral patterns, which can influence the exposure and susceptibility to PM_10_, NO_2_ and SO_2_. These variations in exposure and susceptibility can contribute to the opposite impacts on the influenza epidemic. It is important to note that these are potential reasons for the observed opposite impacts, and further research is needed to fully understand the underlying mechanisms.

Moreover, the direction of the impact of the same air pollutant on influenza epidemics continuously changed over time. For instance, the influence coefficients of O_3_ and CO on the influenza epidemic changed from negative to positive in the western region during the influenza high season (Figs. 7, 9). The concentrations of O_3_ and CO may vary over different time periods. In the early stages of the influenza high season, the concentrations of O_3_ and CO may be relatively low, resulting in a negative impact on the influenza epidemic. However, as time progresses, the concentrations of O_3_ and CO may gradually increase, thereby changing their impact on the influenza epidemic and eventually becoming positive. In addition to changes in O_3_ and CO concentrations, other environmental factors may also affect their impact on the influenza epidemic. For example, factors such as temperature and humidity may vary over time, thereby altering the impacts of O_3_ and CO on the influenza epidemic. However, further investigation is needed to determine the specific mechanism.

Finally, we also found that in some areas, the relationship between air pollution and influenza epidemics may change over time. Cluster 4 (Fig. 12o) was composed of the surrounding areas of Fuzhou, such as Minqing County, Fuqing City and Changle District and other districts and counties, and the impact coefficient changed from negative to positive in 2018. This suggests that local spatial heterogeneity is not static in time but may be dynamic.

By utilizing STWR model, we have identified the complex relationship between air pollution factors and influenza in Fuzhou. The impacts of air pollution on influenza may be dynamic and could vary in different regions and time periods. The research findings emphasize the importance of considering spatiotemporal heterogeneity when studying the relationship between air pollution and influenza. It holds significant value for the development of more effective strategies for preventing and controlling influenza.

### Limitations and future work

However, our current work still has some limitations: (1) The study utilized retrospective observational data, which limits the ability to investigate the direct impact of air pollution on influenza, as well as the ability to obtain specific exposure information from the population regarding air pollution, such as exposure duration. (2) In order to have a more comprehensive understanding of the mechanisms underlying the influenza outbreak in Fuzhou, it is also necessary to consider meteorological factors such as temperature, humidity, and diurnal variations, as well as the economic development level of the region^36^. (3) The current STWR model cannot support multiple scales, which will reduce the reliability of the analysis to a certain extent. (4) The data source used in this study is an infectious diseases reporting information system, which indicates that we can only count information on influenza patients who visit hospitals. 68.74% of the reported cases were pediatric patients under 14 years of age, suggesting that adult influenza patients may have been lost because they did not seek medical care. And the management level at different hospitals may affect the reporting of infectious diseases. (5) We only used data from 2013 to 2019 to exclude the possibility that the outbreak of COVID-19 may have introduced new confounding factors in the studies of influenza.

In future work, it is important to consider the impacts of other factors such as meteorological conditions, economic development levels, and human activities on influenza. Additionally, further investigation can be conducted to determine whether the spatiotemporal heterogeneity in the relationship between air pollution and influenza has changed following the outbreak of COVID-19.

## Conclusions

This study investigated the epidemiological characteristics of influenza in Fuzhou and analyzed the spatiotemporal heterogeneity of the impacts of air pollution on influenza. The following conclusions can be drawn: (1) In Fuzhou, the epidemic of influenza shows a clear trend of high incidence in winter and spring, and the incidence rate has been increasing over the years. The central districts of Fuzhou have a higher incidence rate of influenza. (2) There is a correlation between air pollution and influenza in different county-level regions of Fuzhou. (3) The STWR model outperforms the OLS regression and GWR models and is the optimal regression model. The STWR model, used to study the spatiotemporal heterogeneity of the impacts of air pollution on influenza, helps to understand and identify key air pollutants during different periods of influenza outbreaks. By understanding its spatiotemporal heterogeneity, targeted and effective prevention and control strategies can be developed, providing a scientific basis for the precise management of influenza outbreaks.

In a word, our study provides valuable insights into the spatiotemporal heterogeneity of the impact of air pollution on influenza. STWR model could be a useful method for exploring the spatiotemporal heterogeneity of the impacts of air pollution on influenza in geospatial processes.

### Supplementary Information

Supplementary Figure 1.

Supplementary Figure 2.

Supplementary Figure 3.

Supplementary Figure 4.

Supplementary Figure 5.

Supplementary Legends.

## Data Availability

The influenza case data used and/or analysed during the current study available from the corresponding author on reasonable request. The air pollution data used in this study are freely available on CAQRA (https://doi.org/10.11922/sciencedb.00053). The F-STWR 2.1.5 used in this study are freely available on GitHub^17^.

## Abbreviations

AICc: Ccorrected Akaike information criterion
CAQRA: China Air Quality Reanalysis Data Set
CAS: Chinese Academy of Sciences
CNEMC: Chinese National Center for Environmental Monitoring
CNIDRIS: Chinese Nationwide Notifiable Infectious Diseases Reporting Information System
CO: Carbon monoxide
DTW: Dynamic time warping
GTWR: Geographically and temporally weighted regression
GWR: Geographically weighted regression
IAP: Institute of Atmospheric Physics
ILI: Influenza-like illness
IV: Influenza virus
NO_2_: Nitrogen dioxide
O_3_: Ozone
OLS: Ordinary least squares
PM: Particulate matter
R_2_: R-squared
RSS: Residual sum of squares
SO_2_: Sulfur dioxide
STWR: Spatiotemporal weighted regression
VIF: Variance inflation factor
