# Supplementary figures and images for: Exploring the spatiotemporal relationship between influenza and air pollution in Fuzhou using spatiotemporal weighted regression model
Source: Sci Rep. 2024 Feb 19;14:4116. doi: 10.1038/s41598-024-54630-8 (PMC10876554; doi:10.1038/s41598-024-54630-8)

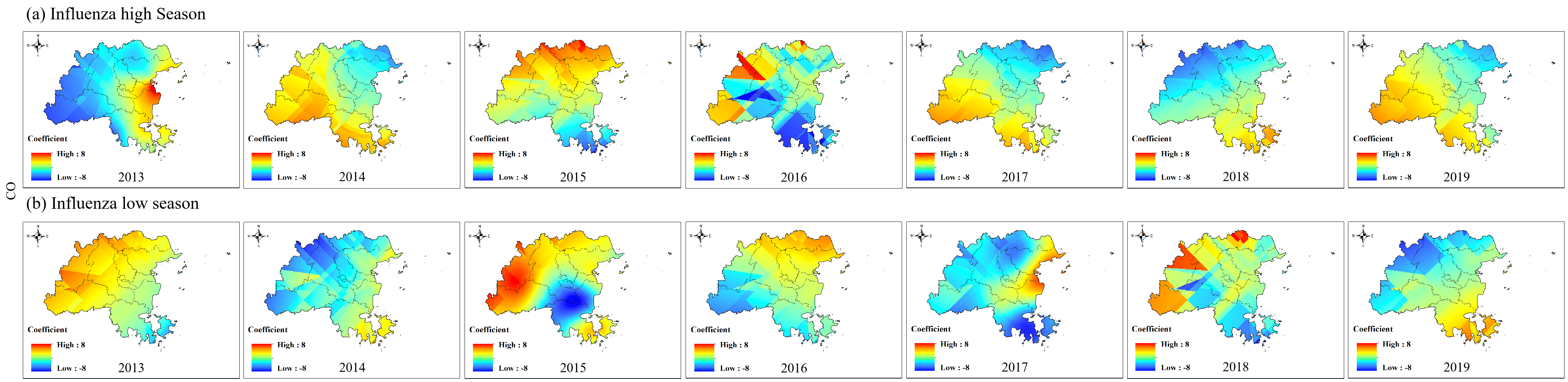

Supplement: Supplementary file 1 — Supplementary Figure 1. [file 41598_2024_54630_MOESM1_ESM.tif]

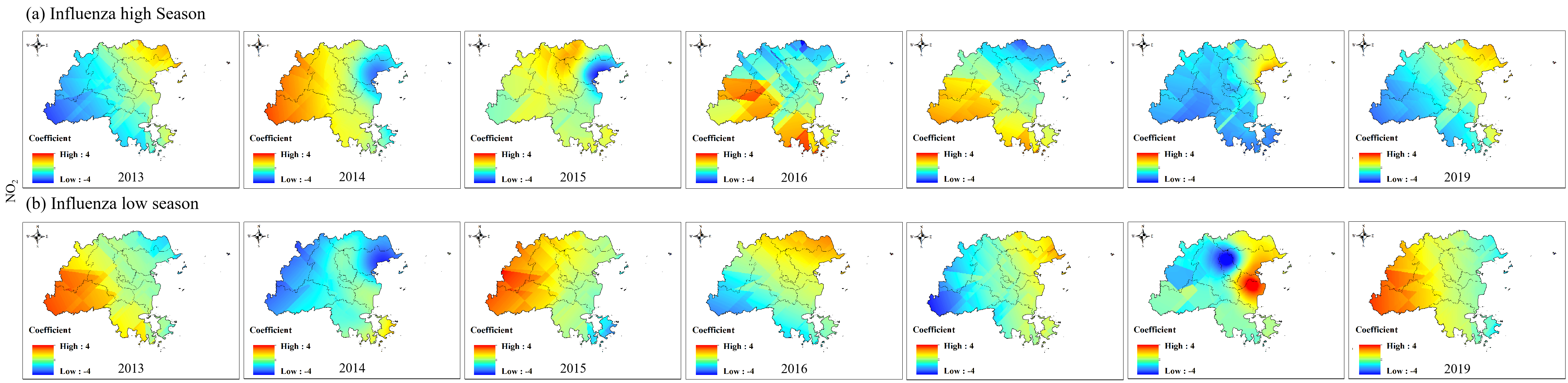

Supplement: Supplementary file 2 — Supplementary Figure 2. [file 41598_2024_54630_MOESM2_ESM.tif]

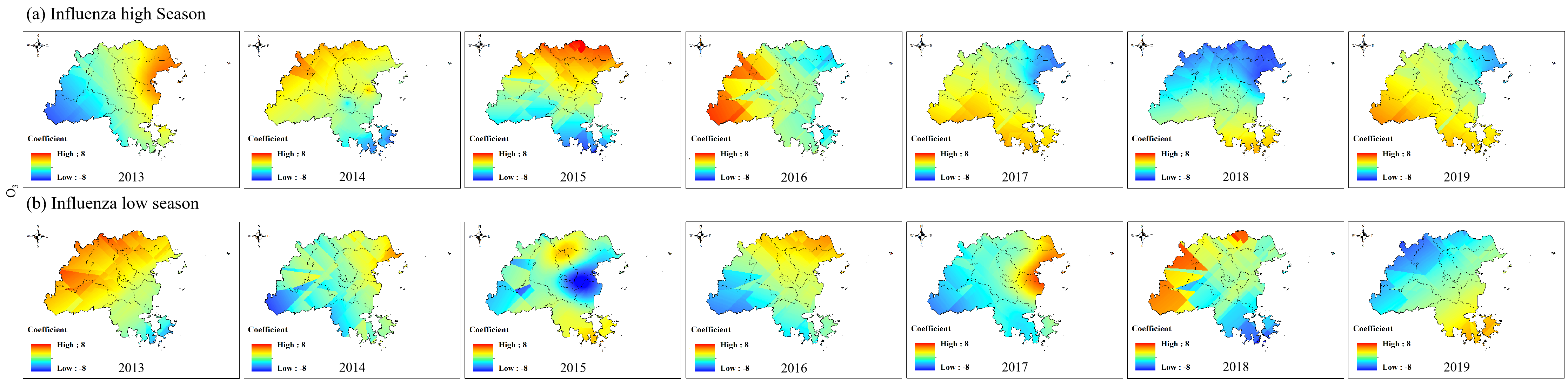

Supplement: Supplementary file 3 — Supplementary Figure 3. [file 41598_2024_54630_MOESM3_ESM.tif]

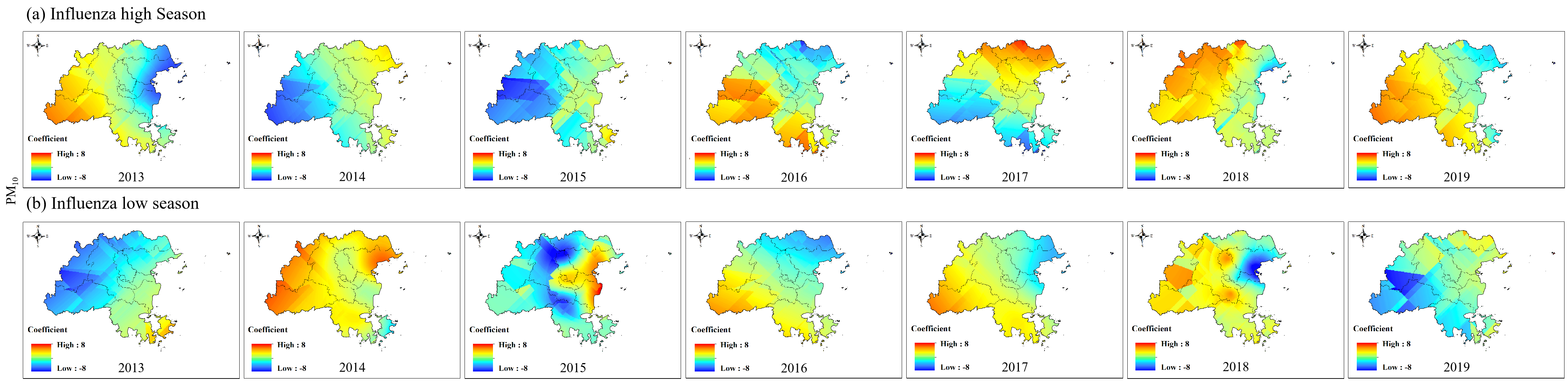

Supplement: Supplementary file 4 — Supplementary Figure 4. [file 41598_2024_54630_MOESM4_ESM.tif]

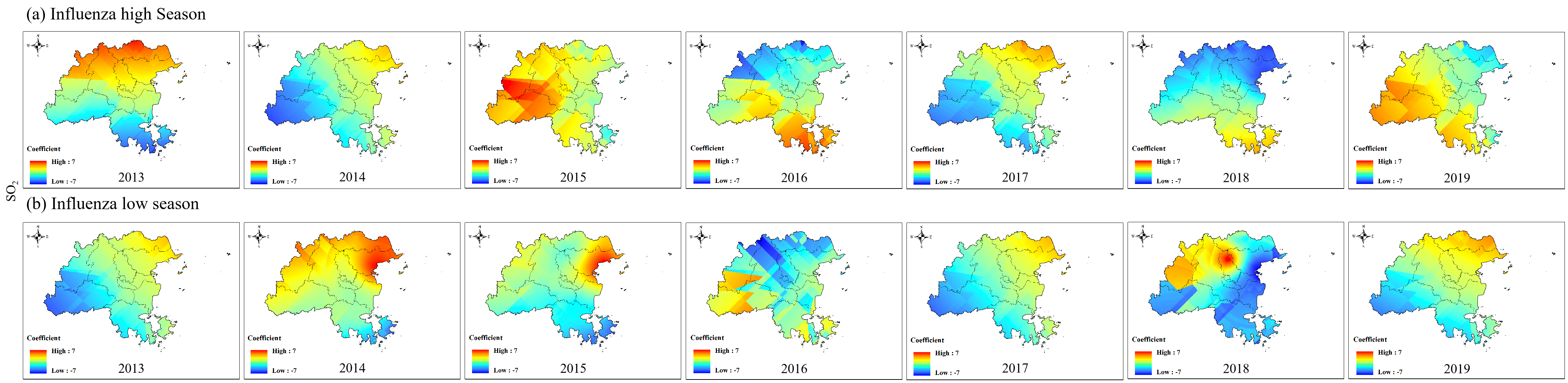

Supplement: Supplementary file 5 — Supplementary Figure 5. [file 41598_2024_54630_MOESM5_ESM.tif]
