# Supplementary material for: Exploring the spatiotemporal relationship between influenza and air pollution in Fuzhou using spatiotemporal weighted regression model
Source: Sci Rep. 2024 Feb 19;14:4116. doi: 10.1038/s41598-024-54630-8 (PMC10876554; doi:10.1038/s41598-024-54630-8)
Supplement: Supplementary file 6 — Supplementary Legends. [file 41598_2024_54630_MOESM6_ESM.docx]

**Supplementary material**

**Figure S1.** Spatial variation coefficient surface of the impact of CO on influenza in Fuzhou, China 2013-2019.

**Figure S2.** Spatial variation coefficient surface of the impact of NO_2_ on influenza in Fuzhou, China 2013-2019.

**Figure S3.** Spatial variation coefficient surface of the impact of O_3_ on influenza in Fuzhou, China 2013-2019.

**Figure S4.** Spatial variation coefficient surface of the impact of PM_10_ on influenza in Fuzhou, China 2013-2019.

**Figure S5.** Spatial variation coefficient surface of the impact of SO_2_ on influenza in Fuzhou, China 2013-2019.
